# Supplementary material for: Neonicotinoid Clothianidin reduces honey bee immune response and contributes to Varroa mite proliferation
Source: Nat Commun. 2020 Nov 18;11:5887. doi: 10.1038/s41467-020-19715-8 (PMC7675992; doi:10.1038/s41467-020-19715-8)
Supplement: Supplementary file 1 — Supplementary Information [file 41467_2020_19715_MOESM1_ESM.pdf]

Supplementary Material

Title

Neonicotinoid Clothianidin reduces honey bee immune response and contributes to *Varroa* mite proliferation

Authors

Desiderato Annoscia, Gennaro Di Prisco, Andrea Becchimanzi, Emilio Caprio, Davide Frizzera, Alberto Linguadoca, Francesco Nazzi & Francesco Pennacchio

Corresponding authors

Francesco Nazzi, francesco.nazzi@uniud.it

Francesco Pennacchio, f.pennacchio@unina.it

Supplementary figures

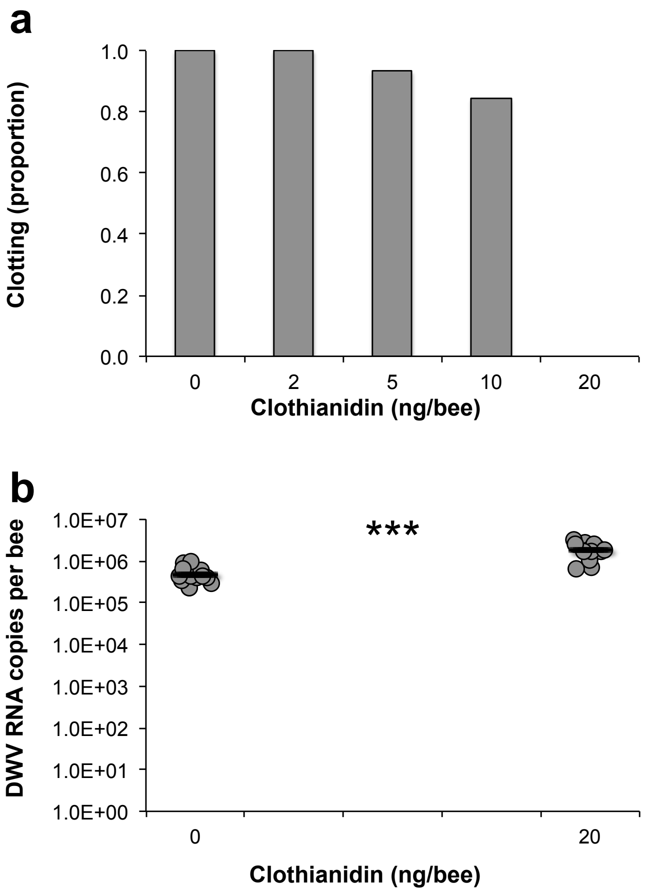


Supplementary Figure 1. **Effect of Clothianidin treatment on clotting and DWV infection level in adult bees. a** Clotting (Spearman's rho=-0.975, n=5, $P<0.05$). **b** DWVinfection level (Mann-Whitney U test: $n_1=15$, $n_2=15$, U=4, $P<0.001$). Three replicates, of five honey bees each, were considered for each experimental condition; the horizontal bars in the scatter-jittered plot represent the sample average.

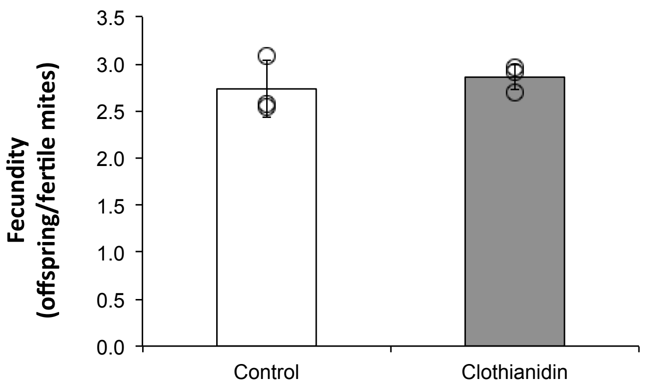


Supplementary Figure 2. **Fecundity (offspring per fertile female) of *Varroa* mites feeding on pupae treated or not with Clothianidin.** The experiment was run in triplicate, for a total of 78 (n=25+17+36 biologically independent mites) and 68 (n=23+14+31 biologically independent mites) individuals for Clothianidin treated and untreated controls, respectively; the offspring per fertile female in each replicate along with the average fecundity and relative standard deviation are reported.

Supplementary tables

Supplementary Table 1. Primers used for qRT-PCR analysis of DWV and immune genes.

| **Transcript** | **Sequence** |
|---|---|
| DWV | F: GCGCTTAGTGGAGGAAATGAA<br>R: GCACCTACGCGATGTAAATCTG |
| Dorsal 1A | F: ACAGGCAGAAGCTGAGAAGC<br>R: TTGCCATCGGATACAAGGAT |
| Amel\102 | F: CAACTCCAGAATTGGAAATAGCA<br>R: TTTGCAATAGGAAAAGCAGTTG |
| β-actin | F: GATTTGTATGCCAACACTGTCCTT<br>R: TTGCATTCTATCTGCGATTCCA |
| rps5 | F: AATTATTTGGTCGCTGGAATTG<br>R: TAACGTCCAGCAGAATGTGGTA |
