# Supplementary material for: Neonicotinoid Clothianidin reduces honey bee immune response and contributes to Varroa mite proliferation
Source: Nat Commun. 2020 Nov 18;11:5887. doi: 10.1038/s41467-020-19715-8 (PMC7675992; doi:10.1038/s41467-020-19715-8)
Supplement: Supplementary file 3 — Reporting Summary [file 41467_2020_19715_MOESM3_ESM.pdf]

nature research

Corresponding author(s): Francesco Nazzi

Last updated by author(s): Sep 3, 2020

Reporting Summary

Nature Research wishes to improve the reproducibility of the work that we publish. This form provides structure for consistency and transparency in reporting. For further information on Nature Research policies, see our Editorial Policies and the Editorial Policy Checklist.

Statistics

For all statistical analyses, confirm that the following items are present in the figure legend, table legend, main text, or Methods section.

n/a	Confirmed	
☐	☒	The exact sample size (*n*) for each experimental group/condition, given as a discrete number and unit of measurement
☒	☐	A statement on whether measurements were taken from distinct samples or whether the same sample was measured repeatedly
☐	☒	The statistical test(s) used AND whether they are one- or two-sided<br>*Only common tests should be described solely by name; describe more complex techniques in the Methods section.*
☒	☐	A description of all covariates tested
☒	☐	A description of any assumptions or corrections, such as tests of normality and adjustment for multiple comparisons
☐	☒	A full description of the statistical parameters including central tendency (e.g. means) or other basic estimates (e.g. regression coefficient) AND variation (e.g. standard deviation) or associated estimates of uncertainty (e.g. confidence intervals)
☐	☒	For null hypothesis testing, the test statistic (e.g. *F*, *t*, *r*) with confidence intervals, effect sizes, degrees of freedom and *P* value noted<br>*Give P values as exact values whenever suitable.*
☒	☐	For Bayesian analysis, information on the choice of priors and Markov chain Monte Carlo settings
☒	☐	For hierarchical and complex designs, identification of the appropriate level for tests and full reporting of outcomes
☒	☐	Estimates of effect sizes (e.g. Cohen's *d*, Pearson's *r*), indicating how they were calculated

Our web collection on statistics for biologists contains articles on many of the points above.

Software and code

Policy information about availability of computer code

Data collection	For the visual analysis of the results of the implantation experiment (Supplementary materials and methods, par. 1.2.), we used GIMP version 2.8 (GNU Image Manipulation Program; www.gimp.org).
Data analysis	All the statistical analyses were made using Excel (version 16.16.20 (200307)); both the data and the analyses can be found in a supplementary data file.

For manuscripts utilizing custom algorithms or software that are central to the research but not yet described in published literature, software must be made available to editors and reviewers. We strongly encourage code deposition in a community repository (e.g. GitHub). See the Nature Research guidelines for submitting code & software for further information.

Data

Policy information about availability of data

All manuscripts must include a data availability statement. This statement should provide the following information, where applicable:

- Accession codes, unique identifiers, or web links for publicly available datasets
- A list of figures that have associated raw data
- A description of any restrictions on data availability

All data are reported in a supplementary data file.

Field-specific reporting

Please select the one below that is the best fit for your research. If you are not sure, read the appropriate sections before making your selection.

[ ] Life sciences [ ] Behavioural & social sciences [x] Ecological, evolutionary & environmental sciences

For a reference copy of the document with all sections, see nature.com/documents/nr-reporting-summary-flat.pdf

Ecological, evolutionary & environmental sciences study design

All studies must disclose on these points even when the disclosure is negative.

Study description	[illegible]tudy aimed at studying if and how the insecticide Clothianidin can interfere with the immune response of honey bees and how [illegible]an in turn affect the reproduction of the parasite Varroa destructor. Therefore, all experiments involved one control group (negative control: untreated) and one (or two, in case more concentrations were tested) treated group to which the insecticide was applied. Each replicate involved a convenient number of bees or mites and were normally repeated three times.
Research sample	[illegible]is case, a research sample is a group of honey bees (Apis mellifera) or a group of mites (Varroa destructor). Given the replicated [illegible]n, based on a large number of individuals per experimental group per replicate, the samples should represent any bee or mite exposed to a given treatment.
Sampling strategy	[illegible]ple size (i.e. number of individual bees/mite used in a single replicate) was roughly pre-determined based on previous [illegible]rience; however, in general, the biggest possible sample size was used in each replicate and at least three replicates of each experiment were carried out on different dates.
Data collection	Data collection involved either the scoring of melanization/encapsulation in bee larvae or measuring the reproduction of individual mites or the quantification of the infection level of bees or the expression of some selected genes. The first two responses were visually assessed the last two were estimated using qRT-PCR.
Timing and spatial scal[illegible]	[illegible]iments on mite reproduction were carried out during the Summer 2016 and 2017. Experiments on the effect of insecticide [illegible]ment on melanization and encapsulation were carried out in Napoli in 2016. Gene expression studies were carried out in Summer 2019.
Data exclusions	No data were excluded from the analysis.
Reproducibility	[illegible]e experiments were replicated a convenient number of times.
Randomization	Allocation of individual bees to the experimental groups was random.
Blinding	In general, the person in charge of scoring the results of an experiment did not know the treatment that was applied to the sample.

Did the study involve field work? [ ] Yes [x] No

Reporting for specific materials, systems and methods

We require information from authors about some types of materials, experimental systems and methods used in many studies. Here, indicate whether each material, system or method listed is relevant to your study. If you are not sure if a list item applies to your research, read the appropriate section before selecting a response.

Materials & experimental systems

n/a	Involved in the study	
[x]	[ ]	Antibodies
[x]	[ ]	Eukaryotic cell lines
[x]	[ ]	Palaeontology and archaeology
[ ]	[x]	Animals and other organisms
[x]	[ ]	Human research participants
[x]	[ ]	Clinical data
[x]	[ ]	Dual use research of concern

Methods

n/a	Involved in the study	
[x]	[ ]	ChIP-seq
[x]	[ ]	Flow cytometry
[x]	[ ]	MRI-based neuroimaging

Animals and other organisms

Policy information about studies involving animals; ARRIVE guidelines recommended for reporting animal research

Laboratory animals	The study did not involve laboratory animals.

Wild animals	The study did not involve wild animals.
Field-collected samples	For all the experiments, honey-bees (Apis mellifera) were collected from the experimental hives of the University of Udine and University of Napoli, which are maintained according to standard beekeeping procedures. The collection of immature bee specimens involved the transfer of brood frames to the lab and the individual picking of mature bee larvae from the combs. For the collection of adult bees, brood frames were transferred to the lab and bees allowed to spontaneously emerge from cells in a net box. Varroa mites (Varroa destructor) were collected from brood cells obtained as above.
Ethics oversight	No ethical approval was required in this case.

Note that full information on the approval of the study protocol must also be provided in the manuscript.
